# The relationship between healthcare providers’ performance regarding women experiencing domestic violence and their demographic characteristics and attitude towards their management

**DOI:** 10.5249/jivr.v10i2.958

**Published:** 2018-07

**Authors:** Nasim Yousefnia, Nafisehsadat Nekuei, Ziba Farajzadegan

**Affiliations:** ^*a*^Students Research Centre, School of Nursing and Midwifery, Isfahan University of Medical Sciences, Isfahan, Iran.; ^*b*^Midwifery & Reproductive Health Department, Nursing and Midwifery Care Research Centre, School of Nursing & Midwifery, Isfahan University of Medical Sciences, Isfahan, Iran.; ^*c*^Community Medicine Department, Isfahan University of Medical Sciences, Isfahan, Iran.

**Keywords:** Healthcare provider, Work performance, Domestic violence, Attitudes, Demographic factors

## Abstract

**Background:**

Domestic violence (DV) can threaten women’s health. Healthcare providers (HCPs) may be the first to come into contact with a victim of DV. Their appropriate performance regarding a victim of DV can decrease its complications. The aim of the present study was to investigate the relationship between HCPs’ performance regarding women experiencing DV, demographic characteristics, and attitudes towards their management.

**Methods:**

In this cross-sectional study, 300 emergency and maternity HCPs were selected using quota random sampling from February to May 2016. All hospitals affiliated to Isfahan University of Medical Sciences (Iran), which are referral centers for DV cases, were selected according to a census. The inclusion criteria included 1 year or more of professional experience and at least 1 encounter with a woman experiencing DV. Data were collected using a researcher-made questionnaire. SPSS was used to analyze the data. Cronbach’s alpha was utilized to assess the reliability of the questionnaire. In order to obtain a general description of the data (variables, mean, and standard deviation), the table of frequencies was designed. Moreover, to determine the relationships between variables, chi-square test was applied.

**Results:**

The results showed that there were no associations between HCPs’ performance regarding DV and their demographic characteristics except their age, professional experience, and economic status. However, there was a significant association between HCPs’ attitudes towards providing services (P=0.017) and their performance regarding women experiencing DV (P less than 0.001).

**Conclusions:**

To improve HCPs’ performance regarding DV, paying attention to other related factors (i.e., training, employing HCPs with high professional experience, and codifying guidelines) is essential. Moreover, elements which result in the creation of positive attitudes and taking care of DV victims should be encouraged.

## Introduction

Physical domestic violence (DV) is any intentional use of physical force by a family member which may lead to the death, disability, and injury of the victim. DV is an important and widespread public health problem. It may lead to physical, sexual, and mental disorders.^1^ The World Health Organization (WHO) has reported that 35% of women worldwide have experienced physical and sexual violence.^2^ Moreover, 45%, 60%, and 24.3% of women in India, Bangladesh, and the United States, respectively, are suffering from physical DV committed by their spouses.^3-5^

Some studies have reported the rate of DV in different parts of Iran such as Marivan (32.9%), Sabzevar (78.1%),^6^ and Tehran (10.7%). It is estimated that the rate of death resulting from DV among women of over 18 years of age is about 36%.^7^ Consequently, with regard to the exclusive role of healthcare providers (HCPs), especially those working in emergency and maternity wards, in identifying and intervening in DV,^8^ they may be the first to come into contact with a victim of DV. Their appropriate performance in terms of measures such as evaluation, intervention, documentation, reference, and follow-up may help reduce damages and improve victims’ circumstances. It may also help decrease DV in Iran as well as the world. ^9-12^

Some studies have been carried out on HCPs’ performance regarding DV, regardless of factors affecting it.^13,14^ Despite the importance of DV and its complications, it seems that no studies have been conducted on HCPs’ performance and factors affecting clinical practice regarding women experiencing DV in Iran. It is assumed that attitudes and demographic characteristics of HCPs may affect their performance when encountering DV victims.^15-18^ Accordingly, the aim of the present study was to investigate the relationship between HCPs’ performance regarding women experiencing domestic violence, their demographic characteristics, and their attitude towards the management of these cases. This study may help to identify some effective factors on HCPs’ performance, and thus, the health of women and mothers.

## Methods 

In the present cross-sectional study, the performance of HCPs working in the emergency and maternity wards of 10 hospitals of Isfahan (Iran) was evaluated from February to May 2016. All hospitals affiliated to Isfahan University of Medical Sciences, which are referral centers for DV cases, were selected according to a census. Out of 796 HCPs, 300 were selected using quota random sampling according to the inclusion criteria. Based on the proportion of the number of eligible HCPs per center relative to the total eligible population, the participants consisted of 42 physicians, 46 midwives, and 212 nurses. The inclusion criteria included 1 year or more of professional experience and at least 1 encounter with a woman experiencing DV. In this research, random sampling method was performed using a random number table; the number of the staff in each hospital was calculated using quota sampling method, based on the amount of staff with inclusion criteria in each center in proportion to the total amount of studied personnel.

The data for the present study were collected through a self-administered questionnaire developed by the researcher. It was based on international guidelines regarding DV and consisted of 3 sections; demographic information (9 items), performances evaluation (5 sections, 35 items), and HCPs’ attitudes towards performance regarding DV victims (i.e., care and support of a DV victim is a professional task) (8 items). The 5 sections of the performance evaluation included assessment (performances regarding physical and mental assessment) (12 items), intervention (performances regarding physical and mental treatment) (7 items), documentation (recording all information on the victim and the services provided for her) (8 items), reference (performances regarding referral of victims to appropriate services) (4 items), and follow-up (following the victim through phone calls or revisits) (4 items). Sections 2 and 3 of the questionnaire were scored based on a 5-point Likert scale (1 = strongly agree, 2 = agree, 3 = no comment, 4 = disagree, and 5 = strongly disagree). Before calculating the sum of the scores, the score of negative questions was reversed. The content validity of the questionnaire was delineated through the use of judgments of experts. To determine quantitative face validity, impact score was used and important items were kept. To determine quantitative content validity, content validity ratio (CVR) (≥ 0.99) and content validity index (CVI) (≥ 0.79) were used. To assess the reliability of the questionnaire, a pilot study was performed on 20 subjects selected from the same study population; these subjects were excluded from the study. The reliability of the questionnaire was confirmed with a Cronbach's alpha of at least 0.75. Finally, the questionnaires were distributed among the participants to be completed in the presence of the researcher, in their spare time with previous coordination.

To analyze the data obtained through the administration of the questionnaire, they were entered into SPSS software (version 20.00, IBM Corporation, Armonk, NY, USA). In order to obtain a general description of the data, descriptive statistics (i.e., mean and standard deviations) were calculated for each subcategory of the questionnaire. Furthermore, chi-square test (χ2) was used to determine the associations of HCPs’ performance with their demographic characteristics and attitudes. Scores of <50%, 50-75%, and > 75% in different sections of the HCP’ performance regarding DV form were, respectively, considered as low, moderate, and high. To assess HCPs’ attitude, it either was considered negative (<50%) or positive (≥ 50%).

## Results

The response rate was 100%, and 300 questionnaires were analysed. The age of the participants ranged from 24 to 50 years (mean = 34.34, SD = 5.98). The majority of the participants (75%) were women with a mean professional experience of 9.10 years (SD = 6.12). All of the participants reported that they had cared for 1-100 cases of DV during their professional experience (mean = 25.29, SD = 23.96). Other demographic characteristics are presented in Table 1. According to the results, the performance of HCPs was evaluated in 5 items (assessment, intervention, documentation, reference, and follow-up). Assessment, intervention, and reference were at moderate levels. Documentation was at a high level, while follow-up was at a low level. The results of this study show significant relationships between some demographic characteristics (i.e., age, professional experience, and economic status) and performance levels of HCPs (P<0.050) (Table 1). Table 2 shows HCPs’ attitude towards performance regarding DV. Based on the results, there was a significant relationship between HCPs’ performance and their attitudes towards performance regarding DV (P<0.001, χ2 = 42.57) (Table 3).

**Table 1:** Association between healthcare providers’ performance and their demographic characteristics (n = 300).

Performance level	Demographic characteristics	Low n (%)	Moderate n (%)	High n (%)	Total n (%)	P-value χ2
**Age (year)**	20-30	29 (9.70)	57 (19.00)	7 (2.30)	93 (31.00)	
	30-40	38 (12.70)	108 (36.00)	16 (5.30)	162 (54.00)	P = 0.031
	> 40	10 (3.30)	24 (8.00)	11 (3.70)	45 (15.00)	χ2 = 10.53
**Gender**	Female	59 (19.70)	139 (46.30)	27 (9.00)	225 (75.00)	P = 0.930
	Male	22 (7.30)	45 (15.00)	8 (2.70)	75 (25.00)	χ2 = 0.14
**Marital status**	Married	51 (17.00)	141 (47.00)	30 (10.00)	222 (74.00)	
	Single	21 (7.00)	46 (15.30)	3 (1.00)	71 (23.70)	P = 0.18
	Other	3 (1.00)	3 (1.00)	1 (0.30)	7 (2.30)	χ2 = 6.30
**Education**	Associate degree	4 (1.30)	5 (1.70)	1 (0.30)	10 (3.30)	
	Bachelor’s degree	23 (7.70)	135 (45.00)	54 (18.00)	212 (70.70)	P = 0.440
	Master’s degree	10 (3.30)	23 (7.70)	3 (1.00)	36 (12.00)	χ2 = 5.87
	Doctorate	6 (2.00)	33 (11.00)	3 (1.00)	42 (14.00)	
**Field of study**	Medicine	9 (3.00)	26 (8.70)	7 (2.30)	42 (14.00)	
	Nursing	56 (18.70)	131 (43.70)	25 (8.30)	212 (70.70)	P = 0.410
	Midwifery	13 (4.30)	31 (10.30)	2 (0.70)	46 (15.30)	χ2 = 4.01
**Economic status**	High	30 (10.00)	51 (17.00)	10 (3.30)	91 (30.30)	
	Moderate	44 (16.70)	122 (40.70)	10 (3.30)	176 (58.70)	P = 0.024
	Low	4 (1.30)	20 (6.70)	9 (3.00)	33 (11.00)	χ2 = 11.15
**Work experience (year)**	1-5	8 (2.70)	48 (16.00)	18 (6.00)	74 (24.70)	
	5-10	15 (5.00)	115 (38.30)	21 (7.00)	151 (50.30)	P = 0.024
	10-15	10 (3.30)	21 (7.00)	2 (0.70)	33 (11.00)	χ2 = 14.68
	> 15	11 (3.70)	25 (8.30)	6 (2.00)	42 (14.00)	
Referred women experiencing physical DV*	<20	45 (15.00)	97 (32.30)	15 (5.00)	157 (52.30)	
	20-50	18 (6.00)	61 (20.30)	8 (2.70)	87 (29.00)	P = 0.480
	50-80	9 (3.00)	21 (7.00)	5 (1.70)	35 (11.70)	χ2 = 5.53
	> 80	5 (1.70)	12 (4.00)	4 (1.30)	21 (7.00)	

*DV: Domestic violence

**Table 2:** Healthcare providers’ attitudes towards performance regarding domestic violence.

Attitude type Items	A/SA* n (%)	D/SD* n (%)	Total n (%)
Helping a woman experiencing DV* is the duty of every human being.	276 (92.00)	24 (8.00)	300 (100)
Care and support for a DV victim is not a professional task.	24 (8.00)	276 (92.00)	300 (100)
In spite of the dissatisfaction of the family of a woman experiencing DV, we should take supportive and therapeutic measures.	280 (93.30)	20 (6.70)	300 (100)
Planning for the treatment and protection of women experiencing DV is a waste of national treasure.	67 (22.30)	233 (77.70)	300 (100)
Receiving high-quality care and support services is the right of every DV victim.	263 (87.70)	37 (12.30)	300 (100)
Physical and psychological evaluation of a woman experiencing DV and her family is necessary.	267 (89.00)	33 (11.00)	300 (100)
Women should not be educated about ways to prevent violence against them.	23 (7.70)	277 (92.30)	300 (100)
Performing therapeutic and supportive measures in a hospital can reduce DV.	252 (84.00)	48 (16.00)	300 (100)

*A/SA: Agree/strongly agree; D/SD: Disagree/strongly disagree

**Table 3:** Association between healthcare providers’ performance and their attitude towards performance regarding domestic violence.

Attitude score		Attitude towards performance regarding DV		Total
Performance level	Positive n (%)	Average n (%)	Negative n (%)	
Low	15 (5.00)	8 (2.70)	3 (1.00)	26 (8.70)
Moderate	33 (11.00)	41 (13.70)	5 (1.70)	79 (26.30)
High	106 (35.30)	76 (25.30)	13 (4.30)	195 (65.00)
Total	154 (51.30)	125 (41.70)	21 (7.00)	300 (100)
P-value/χ2		P<0.001, χ2 = 42.57		

## Discussion

The present study is the first in Iran to evaluate the associations between HCPs’ demographic characteristics and their attitude towards performance regarding DV. The mean number of participants who came into contact with victims of DV was 25.29. No previous research was found to have estimated this number of HCPs encountering DV victims. However, A survey carried out in Hamburg (Germany) reported a range from 1 to 2 cases per year.^15^ Another study conducted in Lebanon reported that physicians’ encountered DV cases with a prevalence of 0.5% to 70% during their professional career. ^17^

According to Table 1, although most HCPs presented an average level of performance, the highest level of performance was observed in HCPs older than 40 years old. The findings of the study showed that there was a significant association between work experience and HCPs’ performance (P<0.05). As it is demonstrated, most HCPs with a high level of performance had a work experience of over 15 years. However, there was no association between the number of DV victims HCPs’ encountered and their performance. The results of the present study are in line with some similar studies^7,15^ in terms of the impact of older age and higher work experience on greater understanding of and sense of responsibility towards social damages. In contrast to the results of some studies showing that gender influenced HCPs’ performance and DV management,^7,15,16^ the findings of the present study showed no association between gender and HCPs’ performance. Accordingly, the participants’ performance in the present study might be related to lack of a DV practical protocol rather than gender differences, so a national protocol can be suggested. 

The outcomes of the current study demonstrated that there was a relationship between HCPs’ economic status and performance. The results of the present study are in line with similar studies showing an association between economic status and healthcare services.^19,20^ In addition, based on the results, there were no relationships between other demographic characteristics and HCPs’ performance regarding DV. Consequently, it seems that most demographic characteristics can affect the performance of HCPs; other factors may be related to their performance.

With regard to the impact of other factors such as the healthcare system, resources and facilities, and leadership and management on HCPs’ performance,^19^ it is necessary to take other factors into consideration to improve HCPs’ performance regarding DV. In order to improve HCPs’ performance, performing more researches, reforming policies, and training and practicing can be suggested.^20^

Additionally, HCPs’ competence, skills, and attitudes should be enhanced.^19^ Based on the results of the present study, HCPs who had a positive attitude towards performance regarding DV presented the highest level of performance; these results are in line with the results of other studies.^7,21^ Nevertheless, the study conducted in Lebanon showed contrasting results.^17^ They displayed that physicians’ attitudes towards the support of battered women were negative. Physicians preferred DV management to be limited to treating physical injuries. Moreover, they believed DV was a private and family issue. Such attitudes, common in Lebanon as well as in Sudan, Pakistan, and Jordan, are culture-bound behaviors.^17^ Furthermore, a study carried out in England showed that despite HCPs’ positive attitudes towards performance regarding DV, their performance was not satisfactory.^18^ This can be due to the lack of self-confidence as well as poor knowledge in managing women experiencing abuse.^18^ Differences in attitudes towards performance regarding DV can result from cultural, social, political, historical, and religious differences^21,22^ as well as knowledge and perception of HCPs regarding DV.^23^ Therefore, to improve the attitudes, knowledge, and performance of HCP, it is necessary to train HCPs^19^ and codify national guidelines on DV. Additionally, the factors creating positive attitudes towards desirable performance regarding DV should be strengthened.

In the present study, the variables influencing HCPs’ performance regarding DV were limited only to their attitudes and demographic characteristics. Due to the proximity of the concepts related to HCPs’ knowledge on DV and the items studied in the questionnaire, it was not possible to evaluate the factor of knowledge. Therefore, it can serve as an interesting area for future research.

## Conclusions

The results of the present study displayed that there were no associations between HCPs’ performance regarding DV and their demographic characteristics except age, professional experience, and economic status. To improve HCPs’ performance regarding DV, training HCPs, employing those with high professional experience, and codifying guidelines on DV for all HCPs with different educational, economic status, age, and hospital positions is recommended. Moreover, factors creating positive attitudes towards performance regarding DV should be enhanced. 

**Acknowledgments**

The authors acknowledge the emergency and maternity wards of hospitals affiliated to Isfahan University of Medical Sciences and their staff for funding the research and the authors are grateful to Saghar Yousefnia for collecting data. They would also like to thank all who cooperated in this study.
